# Upregulation of GNPNAT1 Predicts Poor Prognosis and Correlates With Immune Infiltration in Lung Adenocarcinoma

**DOI:** 10.3389/fmolb.2021.605754

**Published:** 2021-03-25

**Authors:** Wenting Liu, Kaiting Jiang, Jingya Wang, Ting Mei, Min Zhao, Dingzhi Huang

**Affiliations:** Department of Thoracic Oncology, Tianjin Medical University Cancer Institute and Hospital, National Clinical Research Center for Cancer, Tianjin Key Laboratory of Cancer Prevention and Therapy, Tianjin’s Clinical Research Center for Cancer, Tianjin, China

**Keywords:** lung adenocarcinoma, GNPNAT1, prognosis, biomarker, immune infiltration

## Abstract

**Background:**

Glucosamine 6-phosphate *N*-acetyltransferase (GNPNAT1) is a key enzyme in the hexosamine biosynthetic pathway (HBP), which functions as promoting proliferation in some tumors, yet its potential biological function and mechanism in lung adenocarcinoma (LUAD) have not been explored.

**Methods:**

The mRNA differential expression of GNPNAT1 in LUAD and normal tissues was analyzed using the Cancer Genome Atlas (TCGA) database and validated by real-time PCR. The clinical value of GNPNAT1 in LUAD was investigated based on the data from the TCGA database. Then, immunohistochemistry (IHC) of GNPNAT1 was applied to verify the expression and clinical significance in LUAD from the protein level. The relationship between GNPNAT1 and epigenetics was explored using the cBioPortal database, and the miRNAs regulating GNPNAT1 were found using the miRNA database. The association between GNPNAT1 expression and tumor-infiltrating immune cells in LUAD was observed through the Tumor IMmune Estimation Resource (TIMER). Finally, Gene set enrichment analysis (GSEA) was used to explore the biological signaling pathways involved in GNPNAT1 in LUAD.

**Results:**

GNPNAT1 was upregulated in LUAD compared with normal tissues, which was verified through qRT-PCR in different cell lines (*P* < 0.05), and associated with patients’ clinical stage, tumor size, and lymphatic metastasis status (all *P* < 0.01). Kaplan–Meier (KM) analysis suggested that patients with upregulated GNPNAT1 had a relatively poor prognosis (*P* < 0.0001). Furthermore, multivariate Cox regression analysis indicated that GNPNAT1 was an independent prognostic factor for LUAD (OS, TCGA dataset: HR = 1.028, 95% *CI*: 1.013–1.044, *P* < 0.001; OS, validation set: HR = 1.313, 95% *CI*: 1.130–1.526, *P* < 0.001). GNPNAT1 overexpression was correlated with DNA copy amplification (*P* < 0.0001), low DNA methylation (*R* = −0.52, *P* < 0.0001), and downregulation of hsa-miR-30d-3p (*R* = −0.17, *P* < 0.001). GNPNAT1 expression was linked to B cells (*R* = −0.304, *P* < 0.0001), CD4^+^T cells (*R* = −0.218, *P* < 0.0001), and dendritic cells (*R* = −0.137, *P* = 0.002). Eventually, GSEA showed that the signaling pathways of the cell cycle, ubiquitin-mediated proteolysis, mismatch repair and p53 were enriched in the GNPNAT1 overexpression group.

**Conclusion:**

GNPNAT1 may be a potential prognostic biomarker and novel target for intervention in LUAD.

## Introduction

Lung cancer is the leading cause of cancer-related death around the world. In recent years, adenocarcinoma has become the predominant pathological type in lung cancer, accounting for about 40% of all lung cancer patients (Molina et al., 2008). Although the surgery and conventional chemoradiotherapy have improved patient survival, the overall 5-year survival rate was around 15%. Nowadays, the rapid development of molecular detection technology has made cancer treatment more precise and the clinical application of targeted therapy significantly improved the survival rate of patients with positive driver genes. However, there are still many undetected genetic changes that may be functionally important in lung cancer. Therefore, it is urgent to find potential genetic changes to prompt drug development and improve patients’ prognosis and survival.

There is obvious heterogeneity of different cancer patients in the incentive and etiology, but metabolic abnormalities, especially glycometabolism disorder, occur in nearly all tumors (Hanahan and Weinberg, 2011). GNPNAT1 is a crucial enzyme in hexosamine biosynthetic pathway (HBP), one of the essential glucose metabolism pathways branching off from glycolysis. Active metabolism was often occurred in rapidly proliferating tumor cells to promote the progression and metastasis in cancers. Under this circumstances, cancer cells primarily choose to raise the product conversion rate of HBP by increasing the glucose, glutamine, and other nutrients intake, and change cancer-related signaling pathways, such as the Ras (Ying et al., 2012), mechanistic target of rapamycin 2 (mTORC2) (Moloughney et al., 2018), and transforming growth factor-beta 1 (TGF-β1) (Lucena et al., 2016). Studies found that embryonic cells lacking GNPNAT1 exhibited defects in proliferation and adhesiveness, but increased apoptosis ability (Boehmelt et al., 2000). Furthermore, researchers have shown that GNPNAT1 was upregulated in breast cancer and prostate cancer, which were related to tumor proliferation and metastasis (Kaushik et al., 2016; Chokchaitaweesuk et al., 2019). However, its expression and clinical value in lung adenocarcinoma (LUAD) are still unclear.

In this study, we analyzed the differential expression of GNPNAT1 in LUAD and normal tissues and investigated its clinical implication based on the Cancer Genome Atlas (TCGA) database, which was confirmed by immunohistochemistry (IHC) staining. Then, we preliminarily explored the potential mechanism of GNPNAT1 in the development and progression in LUAD. Our research provided a new perspective on the diagnosis and even treatment of LUAD in the future.

## Materials and Methods

### Samples and Cell Lines

The training set samples were obtained from the TCGA database, including 535 LUAD and 59 adjacent normal tissue samples. The transcriptome expression data and clinical information (type: HTSeq–FPKM; time: May 20, 2020) were both downloaded from the TCGA GDC^1^. In the validation set, 116 samples of LUAD and 18 adjacent non-tumor samples were acquired from LUAD patients who underwent surgery between December 2012 and February 2014 at the Tianjin Cancer Institute and Hospital, which was approved by the Ethics Committee of the Tianjin Cancer Institute and Hospital and was consistent with the ethical guidelines of the Helsinki Declaration. All patients signed informed consent, none of who underwent chemotherapy or radiotherapy before surgery. In addition, GSE19188 (Hou et al., 2010), GSE19804 (Lu et al., 2010), GSE31210 (Okayama et al., 2012), and GSE32863 (Selamat et al., 2012) from the GEO database^2^ were used to verify the differential expression at the mRNA level. The baseline information of LUAD patients from the TCGA database and our hospital are shown in Tables 1, 2. The expression levels of GNPNAT1 in different tumor cells and tissues were acquired from the Cancer Cell Line Encyclopedia (CCLE)^3^ (Cerami et al., 2012), TCGA, and Genotype-Tissue Expression (GTEx) databases. Normal lung cell lines (BEAS-2B) and LUAD cell lines (including NCI-H1975, NCI-H358, PC-9, HCC827, and NCI-H1299) in this study were purchased from American Type Culture Collection (ATCC) cell bank.

**TABLE 1:** The clinical characteristics of LUAD patients from TCGA.

Clinical variables	Total (*n* = 522)	%	Clinical variables	Total (*n* = 522)	%
**Age (years)**			**Clinical stage**		
Mean (SD)	65.3 (10.0)	–	Stage I	279	53.45
Median (Min, Max)	66 (33, 88)	–	Stage II	124	23.75
<60	139	26.63	Stage III	85	16.28
≥60	364	69.73	Stage IV	27	5.17
Unknown	19	3.64	Unknown	7	1.34
**Gender**			**Tumor size (cm)**		
Female	280	53.64	T1 (≤3)	172	32.95
Male	242	46.36	T2 (3–5)	281	53.83
**Ethnic origin**			T3 (5–7)	47	9.00
White	392	75.10	T4 (>7)	19	3.64
Non-White	62	11.88	Unknown	3	0.57
Unknown	68	13.03	**Lymph node metastasis**		
**Smoking history**			Negative	335	64.17
Non-smoker	75	14.37	Positive	175	33.52
Current or former smoker	433	82.96	Unknown	12	2.30
Unknown	14	2.68	**Distant metastasis**		
**ECOG-PS**			Negative	353	67.62
≤1	195	37.36	Positive	25	4.79
>1	24	4.60	Unknown	144	27.59
Unknown	303	58.04	**Organ of origin**		
**Cancer status**			Upper lobe	291	55.75
Tumor free	314	60.15	Middle lobe	21	4.02
With tumor	110	21.07	Lower lobe	173	33.14
Unknown	98	18.77	Unknown	37	7.09

*ECOG, Eastern Cooperative Oncology Group.*

**TABLE 2:** The clinical information of LUAD patients in validation set.

Clinical characteristics	Number (116)	%
**Age (year)**		
Median (Min, Max)	59 (31–79)	–
**Gender**		
Male	54	46.55
Female	62	53.45
Smoking history		
Non-smoker	68	58.62
Smoker	48	41.38
**TNM stage**		
Stage I	55	47.41
Stage II	13	11.21
Stage III	48	41.38
**Tumor size**		
T1	69	59.48
T2	41	35.34
T3 + T4	6	5.17
**Lymph node metastasis**		
N0	58	50.00
N1	12	10.34
N2	46	39.66
**ECOG-PS**		
0	50	43.10
1	66	56.90

### Quantitative Real-Time PCR

Trizol reagent was used to extract the total RNA according to the product protocol (Invitrogen, No.15596026). Reverse transcription reaction was carried out to acquire cDNA to prepare for the quantitative real-time PCR with the PrimeScript^TM^ RT Master Mix (TaKaRa, RR036A). qPCR was cycled with the CFX96 quantitative real time gene amplification instrument (Bio-Rad) using 2X SG Fast qPCR Master Mix (Sangon Biotech, No. B639271). Primers for GNPNAT1 are followed from 5′ to 3′: AGGGCCTCTACGGTTCCTGT (F), GTGTTGGGGAAATGGCTGGA (R). HS-ACTB was used as reference primers (Sangon Biotech, B662102-0001). The amplification efficiency was assessed by the standard curve. The experiment was repeated three times.

### Immunohistochemistry Staining in the Validation Set

Fresh LUAD and normal lung specimens after surgery were fixed with formalin solution and embedded in paraffin blocks. Slides were baked to dry in an oven at 70°C overnight. Then, deparaffinized in three changes of xylene, and rehydrated in sequential incubation with 100, 95, 85, and 75% ethanol. Antigen retrieval was performed with pressure cooking in citrate buffer (pH = 6.0) and allowed to cool at room temperature. Quench endogenous peroxidase activity through incubating slides in 3% H_2_O_2_ for 20 min. Subsequently, slides were incubated with the rabbit anti-human GNPNAT1 antibody (Proteintech Cat# 16282-1-AP, RRID: AB_2110243, 1:200) at 4°C overnight. Employing envision secondary antibody for 30 min at room temporary. Slides were stained with 3,3′-diaminobenzidine (DAB) and watched under the microscope. Next, counterstain sections in mayor hematoxylin and rinse slides gently in a distilled water. Transfer slides to a solution containing 1% HCl and 99% ethanol for 6–10 s, and then rinse by water immediately. Eventually, dehydrate by graded ethanol series, incubated in xylene and mount the slides with neutral balsam. GNPNAT1 was localized primarily in the cytoplasm of LUAD cells.

The GNPNAT1 staining intensity was scored across four grades (0, no immune response; 1, weak immune response; 2, moderate immune response; and 3, strong immune response). The percentage of tumor cells positive was evaluated across a range from one to four (1, <25% positive cells; 2, 25–50% positive cells; 3, 50–75% positive cells; and 4, 75–100% positive cells). Each individual intensity score was multiplied by the percentage score to obtain the final IHC score, ranging from 0 to 12.

### Effects of DNA Copy Number Alternations and Methylation Status on GNPNAT1

To observe the potential role of GNPNAT1 in LUAD from the epigenetic perspective, we analyzed the different DNA copy number alterations (CNAs) and methylation of GNPNAT1 expression in LUAD by the cBioPortal database^4^ (Cerami et al., 2012). Moreover, previous studies have shown that abnormal methylation of CpG island was closely related to the occurrence of tumors (Costello et al., 2000; Ehrlich, 2002), so we used MEXPRESS^5^ (Koch et al., 2019), which can visualize DNA methylation, gene expression and clinical data of TCGA, to study the correlation between methylation status of CpG island and its expression. The correlation coefficient |R| > 0.2 and *P* < 0.00001 were considered as having a significant difference in this analysis.

### The Prediction for Upstream miRNAs of Regulating GNPNAT1

DIANA- microT^6^ (Paraskevopoulou et al., 2013) and miRWalk^7^ (Sticht et al., 2018) databases were applied to find out the potential miRNAs regulating GNPNAT1. Then, differential expression miRNAs in LUAD were screened through the OncomiR database^8^ (Wong et al., 2018). The common miRNAs that must be downregulated in LUAD from the TCGA database in the above databases were seen as potential regulatory miRNAs of GNPNAT1 in LUAD. The TargetScanHuman 7.2 database^9^ (Agarwal et al., 2015) was used to predict the possible regions combined with GNPNAT1 3′ UTR binding site.

### The Correlation Between GNPNAT1 and Tumor-Infiltrating Immune Cells in LUAD

To explore the potential immunomodulatory effect of GNPNAT1 in LUAD, the Tumor IMmune Estimation Resource(TIMER) database^10^ (Li et al., 2017) was performed to probe the relevance of GNPNAT1 expression with immune cells (B cells, CD4^+^ T cells, CD8^+^ T cells, neutrophils, macrophages, and dendritic cells). As we all know, tumor cells overexpressed MKI67 and its protein ki67 was the most commonly used as an indicator of tumor proliferation in pathology. Importantly, one recent study has suggested that ki67 overexpression was associated with decreased immune cells and tumor invasion (Mitchell et al., 2019). So, the MKI67 expression in LUAD and normal tissues was observed from the TCGA database and the correlation between GNPNAT1 and MKI67 was evaluated to investigate the role of GNPNAT1 in the immune microenvironment and tumor proliferation.

### Protein-Protein Interaction Network and Gene Set Enrichment Analysis

Gene co-expression analysis has already been proven to be useful in exploring specific genes’ functions. Studies have noticed that co-expressed genes participated in similar biological processes in function (Stuart et al., 2003; Pan et al., 2019). Hence, the co-expressed genes of GNPNAT1 were obtained from the Tissue and Cancer Specific Biological Networks (TCSBN)^11^ (Lee et al., 2018). Then, the acquired genes were imported into Cytoscape^12^ (Shannon et al., 2003) to construct the protein-protein interaction (PPI) network. Gene set enrichment analysis (GSEA) software (version 4.0.3) (Subramanian et al., 2005) was applied to get the relevant signaling pathways involved in GNPNAT1 enrichment genes in LUAD. The annotated gene set of c2.cp.kegg.v6.2.symbols.gmt was chosen to conduct the Kyoto Encyclopedia of Genes and Genomes (KEGG) enrichment analysis. The screening criteria were *P* < 0.05 and false discovery rate (FDR) *q*-value < 0.25, which were deemed as significantly enriched.

### Statistical Analyses

The correlation between GNPNAT1 and LUAD clinical features was evaluated by non-parametric tests. We performed the Mann–Whitney U test for two groups and the Kruskal–Wallis test for no less than three groups, respectively. In the TCGA database, LUAD patients were divided into expression-high and expression-low groups by the median expression of GNPNAT1 (median value = 9.3). In the validation set, the IHC score was seen as GNPNAT1 expression at the protein level. The score no more than seven was regarded as low expression and more than seven scores were the high expression. Kaplan–Meier (KM) analysis was conducted based on the survival time in the low and high group of GNPNAT1 expression. Moreover, univariate and multivariate Cox regression analyses were used to test the correlation between survival time and clinical prognostic indicators and GAPNAT1 expression. Nomograms were constructed based on the independent factors of Cox multivariate analyses in the TCGA set. The concordance index (C-index) and calibration were also assessed to effectively measure the performance of constructed nomograms. The correlation between GNPNAT1 expression and DNA methylation level or immune cell infiltration levels was analyzed by Pearson correlation. R version 3.6.0, GraphPad Prism 7 and SPSS 26.0 were employed for statistical analyses. *P* < 0.05 was considered as statistically significant and all statistical analyses applied bilateral detection (“^∗^”: *P* < 0.05, “^∗∗^”: *P* < 0.01, “^∗∗∗^”: *P* < 0.001, and “^****^”: *P* < 0.0001, ns: insignificant).

## Results

### The Upregulation of GNPNAT1 in LUAD

**FIGURE 1:**
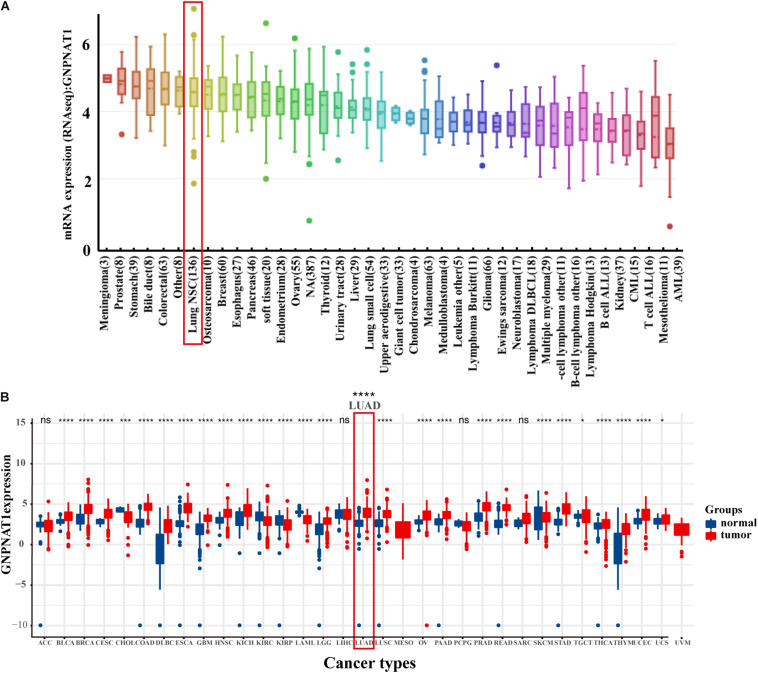
The expression of GNPNAT1 in cancers. **(A)** The mRNA expression level of GNPNAT1 in cancer cells. **(B)** The expression level of GNPNAT1 in different tumor and normal tissues.

**FIGURE 2:**
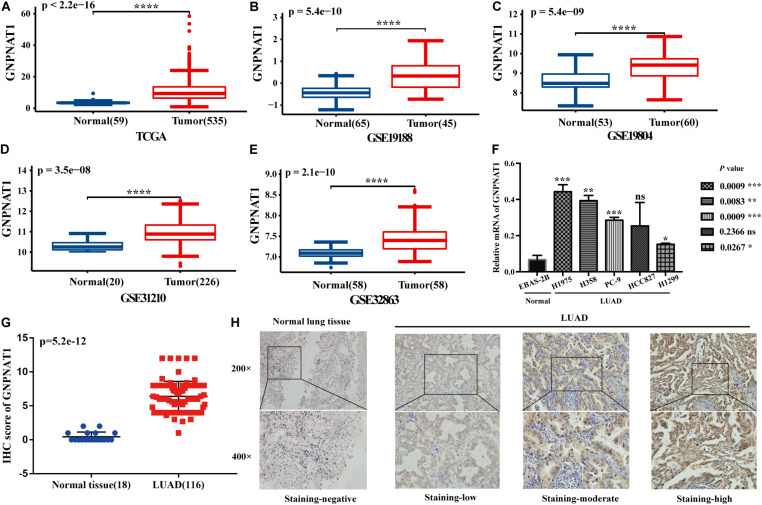
The differential expression of GNPNAT1 in normal tissues and LUAD. **(A)** The differential expression of GNPNAT1 from the TCGA database. The differential expression of GNPNAT1 in GSE19188 **(B)**, GSE19804 **(C)**, GSE31210 **(D)**, and GSE32863 **(E)**. The expression level in different LUAD and normal lung cell lines **(F)**. The differential expression of GNPNAT1 from clinical samples **(G)**. The typical IHC staining results of GNPNAT1 in normal lung tissue and LUAD tissues **(H)**. *P < 0.05, **P < 0.01, ***P < 0.001, and ****P < 0.0001, ns: insignificant.

At the cell level, the CCLE database revealed that GNPNAT1 was up-regulated in NSCLC and other 39 different tumor cells (Figure 1A). At the tissue level, TCGA combined with GTEx database analysis indicated that GNPNAT1 was highly expressed in LUAD and other 27 tumors (*P* < 0.05) (Figure 1B). At the mRNA level, we analyzed 515 patients, including 59 normal tissues and 535 LUAD samples from TCGA. The results unveiled that GNPNAT1 was overexpressed in LUAD (*P* < 0.0001, Figure 2A), which was further certified by GSE19188 (*P* = 5.4e-10), GSE19804 (*P* = 5.4e-9), GSE31210 (*P* = 3.5e-8), and GSE32683 (*P* = 2.1e-10) (Figures 2B–E). To verify this finding, we performed the quantitative real-time PCR assay to compare the relative mRNA level between the normal lung epithelial cell (BEAS-2B) and various LUAD cell lines, including H1975, H358, PC-9, HCC827, and H1299. The results demonstrated that the mRNA level of GNPNAT1 was much higher in LUAD cells than that of BEAS-2B (H1975 vs. EBAS-2B, *P* = 0.0009; H358 vs. EBAS-2B, *P* = 0.0083; PC-9 vs. EBAS-2B, *P* = 0.0009; HCC827 vs. BEAS-2B, *P* = 0.2366; H1299 vs. EBAS-2B, *P* = 0.0267) (Figure 2F). At the protein level, the IHC results of 116 LUAD and 18 normal lung samples showed that GNPNAT1 had an elevated expression level compared with normal lung tissues (*P* = 5.2e-12) (Figures 2G,H). The positive control of GNPNAT1 for IHC is human liver tissue (Supplementary Figure 1A).

### The GNPNAT1 Overexpression Predicts Poor Prognosis in LUAD

**FIGURE 3:**
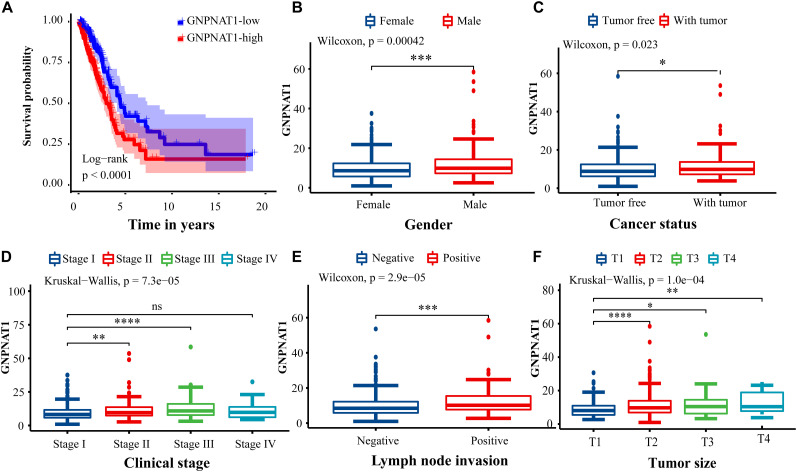
Association of GNPNAT1 expression with clinicopathologic characteristics. **(A)** The survival curve between low and high expression of GNPNAT1 in LUAD. The correlation between GNPNAT1 and clinicopathologic variables involved in gender **(B)**, cancer status **(C)**, clinical stage **(D)**, lymph node metastasis status **(E)**, and tumor size **(F)**. *P < 0.05, **P < 0.01, ***P < 0.001, and ****P < 0.0001, ns: insignificant.

**FIGURE 4:**
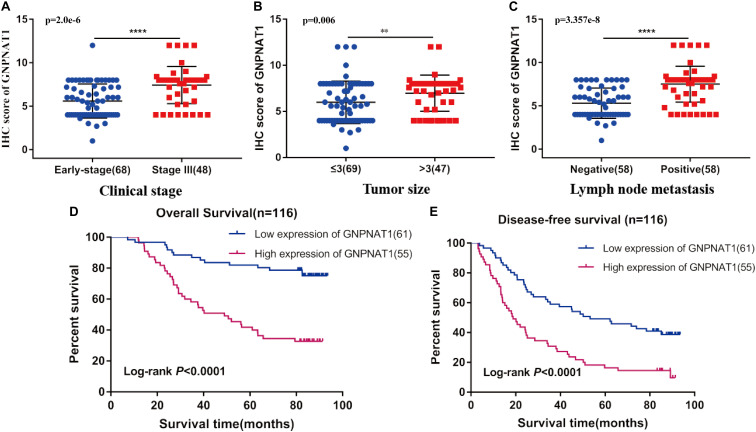
Association of GNPNAT1 expression with clinical features in validation set, including TNM stage **(A)**, tumor size **(B)**, and lymph node metastasis status **(C)**. The Kaplan–Meier curves about the correlation between GNPNAT1 expression and overall survival **(D)** and disease-free survival **(E)**. **P < 0.01 and ****P < 0.0001, ns: insignificant).

**FIGURE 5:**
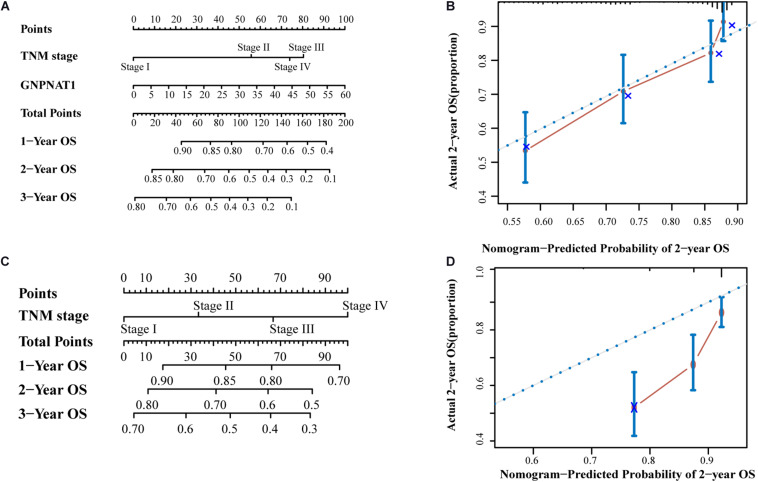
The prognostic nomograms based on GNPNAT1 and TNM stage **(A)** and TNM stage alone **(C)**. The calibration curve of **(A)** nomogram for predicting OS at 2 year **(B)**. The calibration curve of **(C)** nomogram for predicting OS at 2 year **(D)**.

Survival analysis was conducted based on survival time (OS, overall survival; PFS, progression-free survival) and GNPNAT1 expression. The Kaplan–Meier analysis implied that the high expression group of GNPNAT1 had a shortened OS (*P* < 0.0001; Figure 3A), but there was no significant difference on PFS between low and high groups (*P* = 0.084; Supplementary Figure 1B). Correlation analysis displayed that GNPNAT1 was clearly correlated with gender (*P* = 0.00042), clinical stage (stage II vs. stage I, *P* = 0.0067; stage III vs. stage I, *P* = 1.8e-05; stage IV vs. stage I, *P* > 0.05), tumor size (T2 vs. T1, *P* = 3.3e-05; T3 vs. T1, *P* = 0.018; T4 vs. T1, *P* = 0.0064), lymph node metastasis (*P* = 2.9e-5) and patients’ status at the end of follow-up (*P* = 0.023) (Figures 3B–F). There were no significant differences in race (*P* = 0.075), smoking history (*P* = 0.190), age (P = 0.140), the Eastern Cooperative Oncology Group (ECOG) score (*P* = 0.600) and distant metastasis (*P* = 0.640) (Supplementary Figures 1C–G). Multivariate Cox regression analysis proved that GNPNAT1 expression level and TNM stage were both independent prognostic factors in LUAD (GNPNAT1: HR = 1.028, 95% CI: 1.013–1.044, *P* = 0.00021; TNM: HR = 1.542, 95% CI: 1.041–2.284, *P* = 0.0307; Table 3). These results were verified again through the IHC staining in clinical variables, including clinical stage (stage III vs. Early stage, *P* = 2.0e-6), tumor size (No-T1 vs. T1: *P* = 0.006) and lymph node metastasis (positive vs. negative, *P* = 3.4e-8) (Figures 4A–C). Survival analysis and Cox regression analysis further validated the outcomes found from the TCGA database (Figures 4D,E, Table 4, and Supplementary Table 1). To further validate the prognostic value of GNPNAT1 in LUAD, we incorporated GNPNAT1 expression and/or TNM staging to construct predictive nomograms. The calibration plots noted that the nomogram, including the expression of GNPNAT1 (C-index = 0.713), was more precise than another one (C-index = 0.677) (Figure 5 and Supplementary Figure 2).

**TABLE 3:** The Cox regression of clinicopathologic characteristics with the overall survival in LUAD.

Parameters	Univariate analysis			Multivariate analysis		
		
	HR	95%CI	*P*-value	HR	95%CI	*P*-value
Age	1.001	0.982–1.020	0.929	–	–	–
Gender	1.001	0.699–1.434	0.996	–	–	–
Clinical stage	1.645	1.397–1.937	0.000	1.542	1.041–2.284	0.031
Tumor size	1.623	1.310–2.011	0.000	1.194	0.942–1.514	0.142
Distant metastasis	1.681	0.924–3.060	0.089	–	–	–
Lymph nodes invasion	2.721	1.893–3.911	0.000	1.446	0.818–2.556	0.205
GNPNAT1 expression	1.816	1.417–2.328	0.000	1.553	1.013–1.044	0.000

**TABLE 4:** The Cox regression analysis among clinical traits and OS in validation set.

Clinical traits	Univariate analysis			Multivariate analysis		
		
	HR	95%CI	*P*-value	HR	95%CI	*P*-value
Age	0.863	0.496–1.501	0.601	–	–	–
Gender	1.454	0.843–2.510	0.179	–	–	–
Smoking history	1.218	0.704–2.106	0.480	–	–	–
TNM stage	2.089	1.525–2.860	0.000	1.172	0.539–2.547	0.688
Tumor size	1.874	1.087–3.233	0.024	1.040	0.569–1.903	0.898
Lymph node metastasis	4.474	2.378–8.420	0.000	1.993	0.458–8.670	0.358
IHC score of GNPNAT1	1.452	1.273–1.656	0.000	1.313	1.130–1.526	0.000

### DNA Copy Number Amplification and Hypomethylation Led to GNPNAT1 Upregulation in LUAD

**FIGURE 6:**
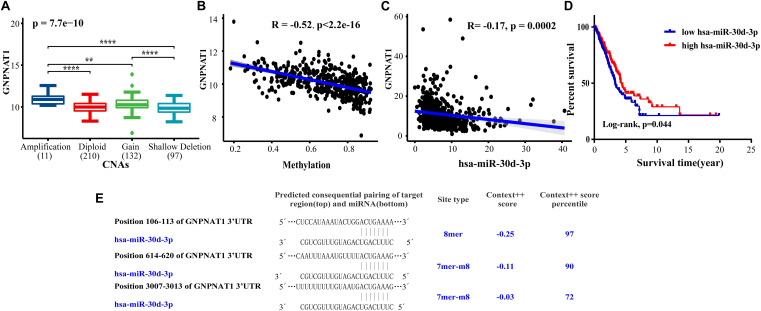
The correlation between GNPNAT1 and some epigenetic factors. The association of GNPNAT1 with its DNA copy alternations **(A)**, methylation **(B)**, and has-miR-30d-3p expression **(C)**. **(D)** The KM curves of the correlation between has-miR-30d-3p expression and overall survival in LUAD based on TCGA data (Cutoff value of high/low group was the median of miRNA expression). **(E)** The predicted target region of GNPNAT1 and has-miR-30d-3p.

Based on the LUAD data from the cBioPortal database containing mRNA, CNAs, and DNA methylation (*n* = 450), we discovered that GNPNAT1 amplification was significantly associated with its mRNA overexpression (Total: P = 7.7e-10; Amplification vs. Diploid, P = 1.9e-05; Amplification vs. Gain, P = 0.002; Gain vs. Shallow Deletion, P = 1.5e-06; Amplification vs. Shallow Deletion, P = 6.2e-06) (Figure 6A). Besides, GNPNAT1 expression also had a closely inverse correlation with DNA methylation (Pearson’s *R* = −0.52, *P* < 0.01) (Figure 6B). We also noticed a negative relationship between GNPNAT1 expression and 10 CpG islands through MEXPRESS (Supplementary Table 2).

### The miR-30d-3p Downregulation Correlated to GNPNAT1 Overexpression in LUAD

We screened sixteen potential target miRNAs by three databases (Supplementary Figure 3 and Table 5). Subsequently, the above miRNAs’ differential expressions in normal and LUAD samples were assessed by the OncomiR database. We discerned that four miRNAs met our research requirements, including hsa-miR-1323, hsa-miR-30a-3p, hsa-miR-30d-3p, and hsa-miR-30d-3p. Multivariate analysis suggested that only hsa-miR-30d-3p was related to pathologic stage (P = 0.031) and tumor size (P = 0.018) (Table 6). Moreover, we discovered that GNPNAT1 expression had certain negative correlation with hsa-miR-30d-3p expression (*R* = −0.170, *P* = 0.0002) (Figure 6C). KM analysis showed that patients with low expression of hsa-miR-30d-3p had a worse prognosis compared with the high expression group (*P* = 0.044) (Figure 6D). The binding site of hsa-miR-30d-3p and GNPNAT1 at 3′UTR was seen in Figure 6E.

**TABLE 5:** Downregulated/upregulated miRNA in LUAD from TCGA.

miRNA	*T*-Test *P*-value	*T*-Test FDR	Upregulated in
hsa-miR-1323	1.77E-02	4.56E-02	Normal
hsa-miR-30a-3p	2.20E-15	1.71E-13	Normal
**hsa-miR-30d-3p**	**1.96E-02**	**4.97E-02**	**Normal**
hsa-miR-138-1-3p	3.43E-02	7.58E-02	Normal
hsa-miR-136-5p	4.05E-06	3.50E-05	Tumor
hsa-miR-148b-3p	2.12E-04	1.02E-03	Tumor
hsa-miR-16-1-3p	1.43E-02	3.94E-02	Tumor
hsa-miR-2355-5p	5.37E-07	5.88E-06	Tumor
hsa-miR-27a-3p	1.20E-02	3.47E-02	Tumor
hsa-miR-27b-3p	1.75E-02	4.56E-02	Tumor
hsa-miR-299-3p	4.99E-02	1.02E-01	Tumor
hsa-miR-335-3p	1.37E-04	6.85E-04	Tumor
hsa-miR-410-3p	1.33E-04	6.73E-04	Tumor
hsa-miR-449a	2.78E-02	6.47E-02	Tumor
hsa-miR-455-3p	5.48E-05	3.25E-04	Tumor
hsa-miR-671-5p	4.89E-08	6.66E-07	Tumor

*Bold Values indicates that hsa-miR-30d-3p was selected by this study.*

**TABLE 6:** The association of hsa-miR-30d-3p with clinical variables in LUAD.

miRNA name	Cancer type	Clinical variables	ANOVA		Multivariate log rank	
				
			*P*-value	FDR	*P*-value	FDR
has-miR-30d-3p	LUAD	Distant metastasis	0.770	0.880	0.005**	0.123
		Pathologic Stage	0.056	0.355	0.031*	0.434
		Tumor size	0.092	0.377	0.018*	0.286
		Gender	0.076	0.347	0.007**	0.140

*P < 0.05, **P < 0.01.

### GNPNAT1 Expression Associated With Immune Cell Infiltration

**FIGURE 7:**
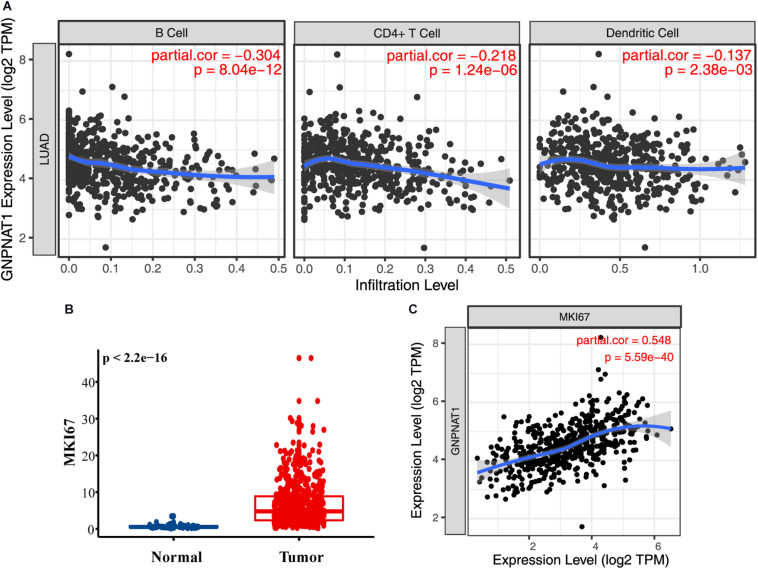
The role of GNPNAT1 in immunomodulation for LUAD. **(A)** The association between GNPNAT1 expression and tumor infiltration immune cells. **(B)** The expression level of MKI67 in LUAD and normal tissues from TCGA. **(C)** The correlation of GNPNAT1 and MKI67 based on TIMER database.

Tumor IMmune Estimation Resource results noted that GNPNAT1 expression was negatively correlated with B cells (*R* = −0.304, *P* = 8.04e-12), CD4^+^ T cells (*R* = −0.218, *P* = 1.24e-06), and dendritic cells (*R* = −0.137, *P* = 0.002) (Figure 7A). Additionally, MKI67 was upregulated in LUAD (*P* < 0.01), and we also observed a closely positive relation between GNPNAT1 and MKI67 (Pearson *R* = 0.548, *P* < 0.01) (Figures 7B,C).

### PPI and GSEA

**FIGURE 8:**
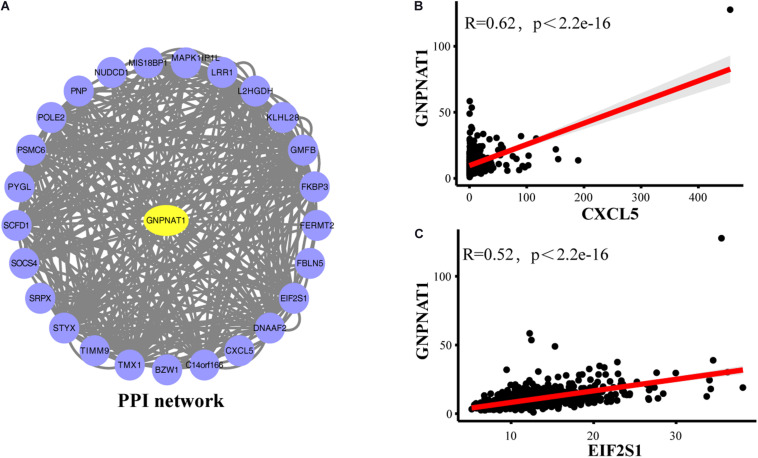
The PPI network of GNPNAT1 in LUAD. **(A)** The PPI network was constructed by database. The correlation between GNPNAT1 and CXCL5 **(B)**/EIF2SI **(C)**.

**FIGURE 9:**
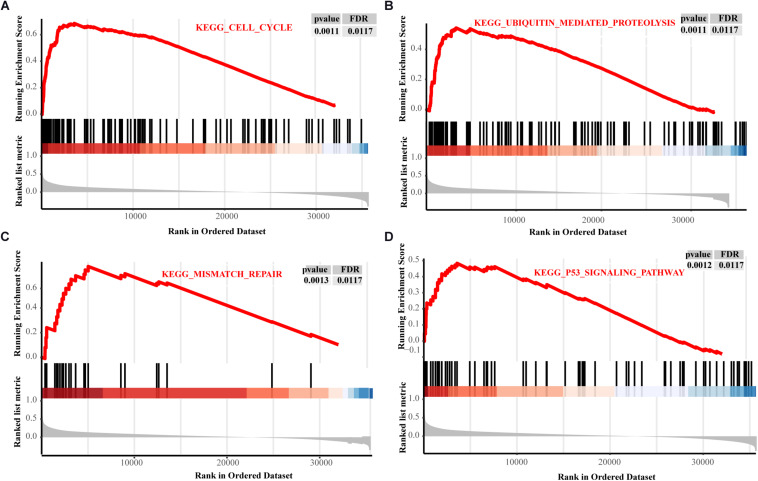
The GSEA results about GNPNAT1 in LUAD from TCGA. The signaling pathways of the cell cycle **(A)**, ubiquitin mediated proteolysis **(B)**, mismatch repair **(C)**, and p53 signaling pathway **(D)**.

The PPI network showed that GNPNAT1 had a positive correlation with 25 genes (*P* < 0.05) (Figure 8A), among which CXCL5 and EIF2S1 had a relatively stronger correlation with GNPNAT1 (*R*_CXCL5_ = 0.62, *P* < 2.2e-16; *R*_EIF2S1_ = 0.52, *P* < 2.2e-16) (Figures 8B,C and Supplementary Table 3). Besides, GSEA revealed that upregulated GNPNAT1 was mainly enriched in the cell cycle, ubiquitin-mediated proteolysis, mismatch repair, and p53 signaling pathways (Figures 9A–D).

## Discussion

Glucose metabolism disorder is a common feature in many cancers, which has become the focused area of anti-tumor drug research (Abdel-Wahab et al., 2019). The principle that tumors have increased demand for glucose has already been widely used by FDG-PET/CT to focus on lesions (Gambhir, 2002). HBP is a branch of glycometabolism in which only about 3–5% of glucose in tumor cells can enter (Akella et al., 2019), but this pathway is the hub of energy metabolism that links glucose, lipid, and protein metabolism. Except for increasing glucose consumption, tumor cells also enhanced the demand for glutamine, an essential substrate for HBP. Studies have found that HBP was involved in the energy metabolism of lung cancer, prostate cancer, and other tumors (Itkonen et al., 2015; Taparra et al., 2018). Furthermore, the final product of HBP, uracil diphosphate *n*-acetylglucosamine (UDP-GlcNAc), is an essential cell signal regulator and contributes to tumor growth. Also, researchers observed that GNPNAT1 was significantly up-regulated in prostate cancer (Kaushik et al., 2016), and GNPNAT1 low expression led to reduced proliferation of tumor cells in lung cancer receiving chemotherapy (Zhao et al., 2017). However, there has no study regarding the expression and clinical role of GNPNAT1 in LUAD.

In this study, our results indicated that GNPNAT1 was increased in various tumors, including LUAD, and its overexpression was related to advanced staging, lymph node metastasis, and poor prognosis. Additionally, multivariate Cox analysis revealed that GNPNAT1 was an independent prognostic factor in LUAD. The nomograms suggested that GNPNAT1 may play a potential role in clinical diagnosis and prognosis assessment.

Genomic instability is another critical trait of tumors (Hanahan and Weinberg, 2011), so we probed the possible mechanism of GNPNAT1 overexpression in LUAD from epigenetics. GNPNAT1 was located at chromosome 14q22.1, and our study illustrated that the DNA amplification of GNPNAT1 had a close correlation with its overexpression (*P* < 0.05), which was consistent with one research that the region of 14q22-q24 was significantly amplified in prostate cancer (Bernardino et al., 1997). Furthermore, DNA hypomethylation can also induce genomic instability and play a pro-oncogenic role (Esteller and Herman, 2002), which has been regarded as a prognostic marker in cancers, such as lung cancer and bladder cancer (Moore et al., 2008; Pfeifer and Rauch, 2009). We also noticed that upregulated GNPNAT1 had a positive relation with DNA hypomethylation. Further analysis found that there was a negative correlation between GNPNAT1 overexpression and multiple sites on CpG island, which implied GNPNAT1 might protect tumors from damage through DNA copy number amplification and CpG island hypomethylation.

It was generally reckoned that miRNAs involved multiple tumorigenesis processes and the primary mechanism was that miRNAs could cause the abnormal expression of target genes (Esquela-Kerscher and Slack, 2006). In recent years, the function of miRNA, LncRNA and other non-coding RNAs (ncRNAs) have been gradually established in tumor metabolism. Increasing evidence showed that ncRNAs could directly target glucose transport or consumption or indirectly regulate cancer-related signaling pathways to change glucose metabolism and affect tumor occurrence and development (Shankaraiah et al., 2018). Therefore, we investigated the possible mechanism of GNPNAT1 over-expression in LUAD from its upstream, and results showed that hsa-miR-30d-3p was significantly down-regulated in LUAD and associated with some clinical features. Researchers have reported that hsa-miR-30d-3p was down-regulated in lung cancer and its down-regulation promoted tolerance to EGFR-targeted drug in lung cancer patients (Pan et al., 2019). Additionally, correlation analysis demonstrated that hsa-miR-30d-3p was conversely associated with GNPNAT1 expression and patients with low hsa-miR-30d-3p expression had a poorer prognosis. The above results manifested the down-regulation of hsa-miR-30d-3p may promote GNPNAT1 overexpression by certain pathways, which led to a worse prognosis among LUAD patients.

Tumor microenvironment, the basis of tumor growth and development, was infiltrated by immune cells which, to some extent, could determine the effect of immunotherapy on patients (Li et al., 2016). According to some studies, abnormal glucose metabolism resulted in the accumulation of lactic acid in the tumor microenvironment, promoting tumor growth and invasion by weakening T cells’ activation and migration of monocytes (Gottfried et al., 2006; Fischer et al., 2007). A report at the 20th World Lung Cancer Congress (WCLC) also pointed out that the glucose transporter (GLUT3/GLUT1 ratio) could predict the immunotherapy response of lung cancer (Na et al., 2019). As we know, GNPNAT1 and GLUT1 were both involved in the glucose metabolism pathway, and they had a positive correlation on expression level. Thus, we explored the correlation between GNPNAT1 expression and immune cells through the TIMER database. The analysis revealed that GNPNAT1 had a converse correlation with the infiltration of B cells, CD4^+^ T cells, and dendritic cells, which all had an anti-tumor effect in NSCLC (Inoshima et al., 2002; Germain et al., 2014; Bruno et al., 2017). GNPNAT1 is one of the key enzymes of HBP process, the end product of which is an essential substrate of glycosylation. Studies suggested that immune cells could express different types of glycosylation-dependent lectin receptors, which affect the function of antigen-presenting cell (APC) and inhibit T-cell activity to promote immune escape by binding to tumor cell surface glycoproteins or glycolipids (RodrÍguez et al., 2018; Sasawatari et al., 2020). Hence, we speculated that the overexpression of GNPNAT1 might affect the function of tumor-infiltrating immune cells by regulating glycosylation modifications. We also noticed a positive correlation between GNPNAT1 and MKI67. Importantly, a previous study has reported that ki67 was associated with decreased immune cells in NSCLC (Mitchell et al., 2019), suggesting that GNPNAT1 may indirectly affect immune function through interacting with MKI67.

Since there were no studies to explore the biological functions of GNPNAT1 overexpression in tumors, we constructed the PPI network which found CXCL5 and EIF2S1 both had a relatively stronger correlation with GNPNAT1. Previous studies have pointed out that CXCL5 was closely related to the invasion and progression of lung cancer (Saintigny et al., 2013), and EIF2S1 could activate autophagy and promote the occurrence of tumors (Dey et al., 2013), so it was reasonable to conjecture that GNPNAT1 might play a synergistic role with these co-expression genes in promoting tumor proliferation.

O-GlcNAcylation, one type of post-translational modification in HBP, was prevalent in tumors, and its high expression level was found to cause p53 instability and promote ubiquitin-mediated proteasomal degradation, thereby leading to resistance to cisplatin-induced apoptosis in lung cancer (Luanpitpong et al., 2017). Recent evidence showed that O-GlcNAcylation promoted mutant KRAS-induced lung tumorigenesis (Taparra et al., 2018). GSEA results also reminded us that GNPNAT1-high group was mainly enriched in the cell cycle, ubiquitin-mediated proteolysis, mismatch repair and p53 signaling pathway, in line with the above study, which proved our GSEA enrichment results were meaningful.

Meanwhile, considering that the partial analysis results of this study are obtained through mining sequencing data, there are several limitations in our study. Firstly, the association between GNPNAT1 and tumor-infiltrating immune cells in lung cancer was acquired *via* cancer database and bioinformatics analysis methods, it is necessary for researchers to further explore the immune regulatory function of GNPNAT1 *in vivo* and *in vitro* experiments. Secondly, the signaling pathways analyzed in this study were discovered based on data mining, experiments were also needed to verify its causal relationship in lung cancer. Finally, clinical samples in the validation set were relatively small, and we will expand the sample size for analysis. In the future, we will investigate the effect of GNPNAT1 on lung cancer cells through invasion and migration experiments *in vitro*, verify the regulatory relationship between GNPANT1 and miRNA-30d-3p and finally propose to explore the effect of GNPNAT1 on lung cancer through animal models.

In conclusion, GNPNAT1 may serve as a prognostic biomarker and indirectly involve in immune regulation for LUAD. Its upregulation might be regulated by DNA copy number amplification, hypomethylation, and miR-30d-3p down-regulation.

## Data Availability Statement

The datasets presented in this study can be found in online repositories. The names of the repository/repositories and accession number(s) can be found in the article.

## Ethics Statement

The studies involving human participants were reviewed and approved by Tianjin Medical University Cancer Institute and Hospital. The patients/participants provided their written informed consent to participate in this study. Written informed consent was obtained from the individual(s) for the publication of any potentially identifiable images or data included in this article.

## Author Contributions

WL designed the study, performed the experiment, analyzed the data, and wrote the first draft. KJ participated in the experiment and revised the manuscript. JW assisted in analyzing and revising the manuscript. TM and MZ performed some data analyses. DH directed and supervised the whole work. All participants have read and approved for this manuscript.

## Conflict of Interest

The authors declare that the research was conducted in the absence of any commercial or financial relationships that could be construed as a potential conflict of interest.

## Footnotes

1 https://portal.gdc.cancer.gov/

2 https://www.ncbi.nlm.nih.gov/geo/

3 https://portals.broadinstitute.org/ccle

4 https://www.cbioportal.org/

5 https://mexpress.be/

6 http://www.microrna.gr/webServer

7 http://mirwalk.umm.uni-heidelberg.de/

8 http://oncomir.org/oncomir/index.html

9 http://www.targetscan.org/

10 https://cistrome.shinyapps.io/timer/

11 http://inetmodels.com

12 https://cytoscape.org/
