# Supplementary material for: Upregulation of GNPNAT1 Predicts Poor Prognosis and Correlates With Immune Infiltration in Lung Adenocarcinoma
Source: Front Mol Biosci. 2021 Mar 25;8:605754. doi: 10.3389/fmolb.2021.605754 (PMC8027087; doi:10.3389/fmolb.2021.605754)
Supplement: Supplementary file 1 [file Data_Sheet_1.docx]

**Supplementary Tables**

**Table S1** The Cox regression analysis among clinical traits and DFS in validation set

| **Clinical traits** | **Univariate analysis** | | | **Multivariate analysis** | | |
| --- | --- | --- | --- | --- | --- | --- |
|  | **HR** | **95%CI** | ***P*-value** | **HR** | **95%CI** | ***P*-value** |
| Age | 0.830 | 0.537-1.282 | 0.401 |  |  |  |
| Gender | 1.054 | 0.537-1.616 | 0.811 |  |  |  |
| Smoking history | 0.858 | 0.553-1.333 | 0.496 |  |  |  |
| TNM stage | 2.046 | 1.609-2.602 | **0.000** | 1.569 | 0.839-2.933 | 0.158 |
| Tumor size | 2.428 | 1.573-3.745 | **0.000** | 1.545 | 0.965-2.476 | 0.070 |
| Lymph node metastasis | 3.596 | 2.268-5.701 | **0.000** | 1.225 | 0.458-8.670 | 0.737 |
| IHC score of GNPNAT1 | 1.452 | 1.126-1.340 | **0.000** | 1.109 | 1.130-1.526 | 0.103 |

**Table S2** Correlation between the expressions of GNPNAT1 and its methylation values

| **Gene** | **CpG site** | **Pearson r** | ***P*-Value** | |
| --- | --- | --- | --- | --- |
| **GNPNAT1** | cg15418809 | -5.24E-01 | **5.90E-27** | *** |
|  | cg04150276 | -4.87E-01 | **2.89E-24** | *** |
|  | cg05225684 | -4.54E-01 | **7.40E-22** | *** |
|  | cg16764848 | -2.81E-01 | **6.51E-10** | *** |
|  | cg02247430 | -2.74E-01 | **1.49E-09** | *** |
|  | cg11045303 | -2.44E-01 | **8.08E-08** | *** |
|  | cg17417600 | -2.27E-01 | **6.22E-07** | *** |
|  | cg18554485 | -2.24E-01 | **8.14E-07** | *** |
|  | cg14117643 | -2.22E-01 | **1.01E-06** | *** |
|  | cg12227762 | -2.10E-01 | **3.86E-06** | *** |
|  | cg26228241 | -1.85E-01 | 4.99E-05 |  |
|  | cg18214930 | 1.34E-03 | 9.77E-01 |  |
|  | cg02867991 | NA | NA |  |
|  | cg18413572 | NA | NA |  |
|  | cg20395072 | NA | NA |  |

**Table S3** The correlation between GNPNAT1 and co-expression genes

| **Gene** | **Co-expression genes** | **Pearson R** | ***P*-value** |
| --- | --- | --- | --- |
| **GNPNAT1** | LRR1 | 0.50 | p < 2.2e-16 |
|  | SRPX | 0.50 | p < 2.2e-16 |
|  | TMX1 | 0.49 | p < 2.2e-16 |
|  | GMFB | 0.48 | p < 2.2e-16 |
|  | L2HGDH | 0.47 | p < 2.2e-16 |
|  | STYX | 0.47 | p < 2.2e-16 |
|  | PSMC6 | 0.46 | p < 2.2e-16 |
|  | FERMT2 | 0.45 | p < 2.2e-16 |
|  | POLE2 | 0.45 | p < 2.2e-16 |
|  | NUDCD1 | 0.44 | p < 2.2e-16 |
|  | PYGL | 0.44 | p < 2.2e-16 |
|  | FBLN5 | 0.43 | p < 2.2e-16 |
|  | DNAAF2 | 0.42 | p < 2.2e-16 |
|  | TIMM9 | 0.40 | p < 2.2e-16 |
|  | FKBP3 | 0.39 | p < 2.2e-16 |
|  | MIS18BP1 | 0.38 | p < 2.2e-16 |
|  | BZW1 | 0.37 | p < 2.2e-16 |
|  | KLHL28 | 0.35 | p < 2.2e-16 |
|  | SOCS4 | 0.35 | p < 2.2e-16 |
|  | MAPK1IP1L | 0.33 | p < 2.2e-16 |
|  | SCFD1 | 0.33 | 2.10E-15 |
|  | PNP | 0.31 | 1.00E-13 |

**Supplementary Figures captions**

**Supplementary Figure S1.**  The positive control of GNPNAT1 for IHC (**A**). The Kaplan-Meier curves about the correlation between GNPNAT1 expression and progression-free survival (**B**). Association of GNPNAT1 with other clinical traits in LUAD **(C-G)**.

**Supplementary Figure S2.**  The calibration plots of nomograms based on GNPNAT1 and TNM stage, and TNM stage alone for predicting OS at 1 year **(A** and **C)** and 3 years **(B** and **D)** in LUAD patients.

**Supplementary Figure S3.**  The venn diagram based on three miRNA databases.
