# Supplementary figures and images for: Upregulation of GNPNAT1 Predicts Poor Prognosis and Correlates With Immune Infiltration in Lung Adenocarcinoma
Source: Front Mol Biosci. 2021 Mar 25;8:605754. doi: 10.3389/fmolb.2021.605754 (PMC8027087; doi:10.3389/fmolb.2021.605754)

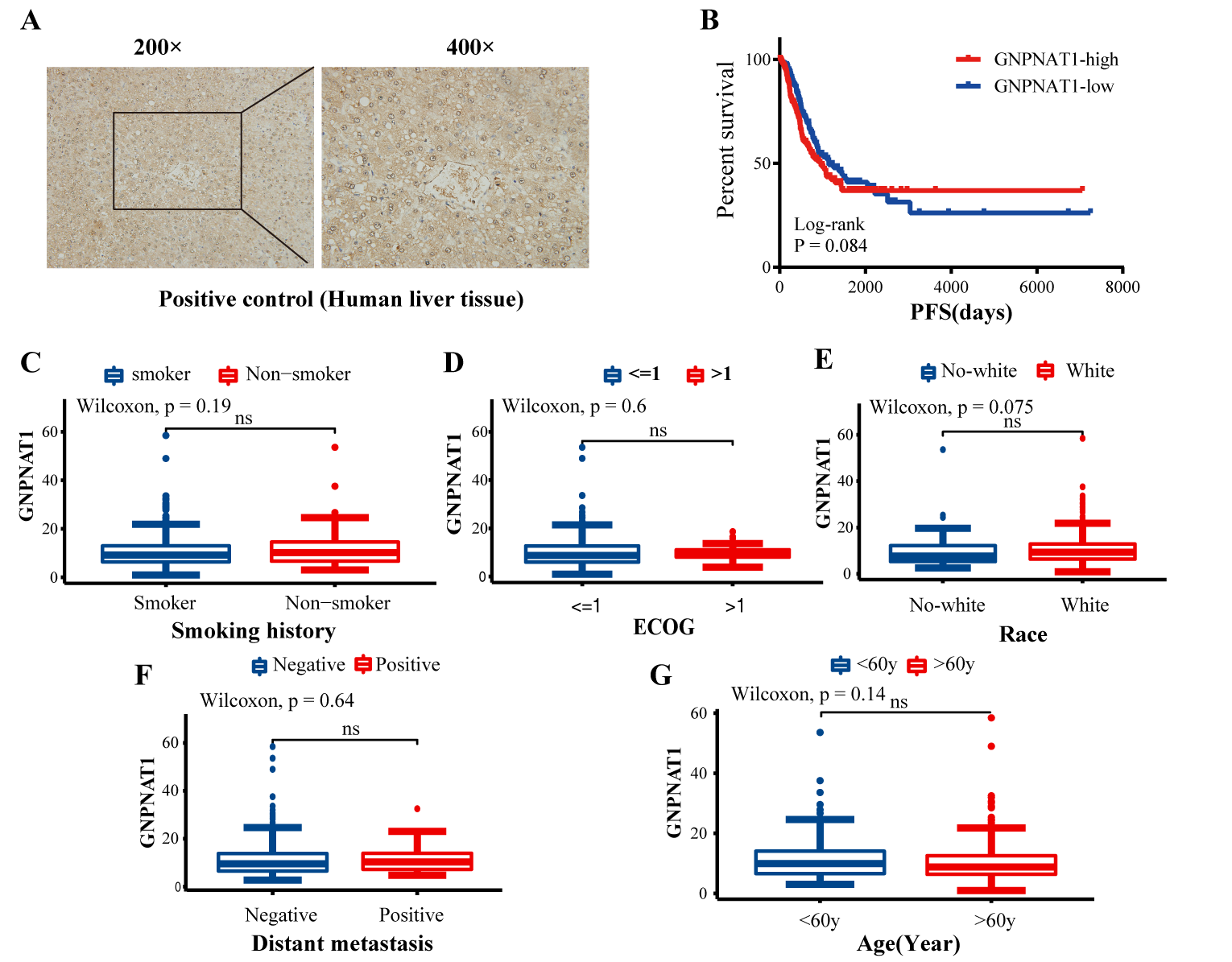

Supplement: Supplementary file 3 [file Image_1.TIF]

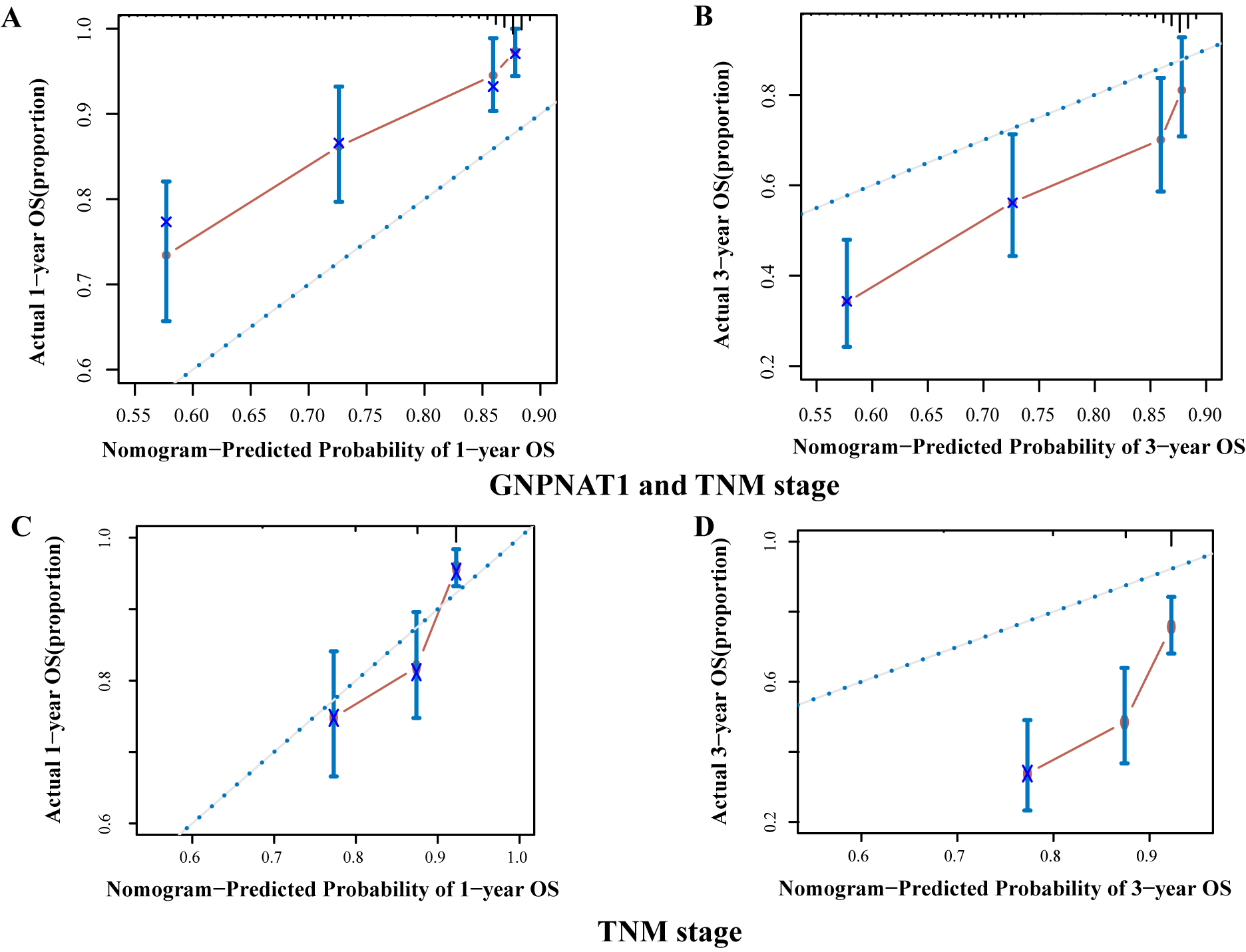

Supplement: Supplementary file 4 [file Image_2.TIF]

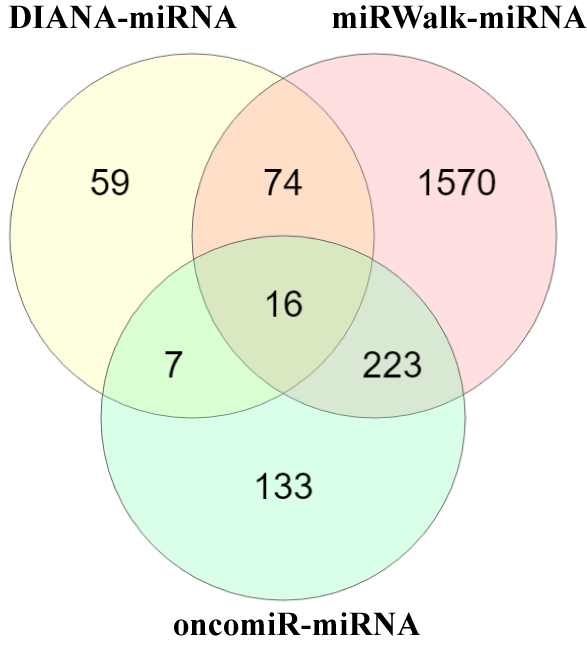

Supplement: Supplementary file 5 [file Image_3.TIF]
